# Unified molecular approach for spatial epigenome, transcriptome, and cell lineages

**DOI:** 10.1073/pnas.2424070122

**Published:** 2025-04-18

**Authors:** Yung-Hsin Huang, Julia A. Belk, Ruochi Zhang, Natasha E. Weiser, Zachary Chiang, Matthew G. Jones, Paul S. Mischel, Jason D. Buenrostro, Howard Y. Chang

**Affiliations:** ^a^Department of Dermatology, Center for Personal Dynamic Regulomes and Program in Epithelial Biology, Stanford University School of Medicine, Stanford, CA 94305; ^b^Broad Institute of Massachusetts Institute of Technology and Harvard, Cambridge, MA 02142; ^c^Department of Stem Cell and Regenerative Biology, Harvard University, Cambridge, MA 02138; ^d^Gene Regulation Observatory, Broad Institute of Massachusetts Institute of Technology and Harvard, Cambridge, MA 02142; ^e^Department of Pathology, Stanford University, Stanford, CA 94305; ^f^Sarafan Chemistry, Engineering, and Medicine for Human Health, Stanford University, Stanford, CA 94305; ^g^Howard Hughes Medical Institute, Stanford University, Stanford, CA 94305

**Keywords:** spatial genomics, spatial multiomics, chromatin accessibility, cell lineages

## Abstract

Spatial epigenomics and multiomics can provide fine-grained insights into cellular states but their widespread adoption is limited by the requirement for bespoke slides and capture chemistries for each data modality. Here, we present SPatial assay for Accessible chromatin, Cell lineages, and gene Expression with sequencing (SPACE-seq), a method that utilizes polyadenine-tailed epigenomic libraries to enable facile spatial multiomics using standard whole transcriptome reagents. Applying SPACE-seq to a human glioblastoma specimen, we reveal the state of the tumor microenvironment, extrachromosomal DNA copy numbers, and identify putative mitochondrial DNA variants.

## Significance

The spatial organization of cells within tissues plays a critical role in maintaining normal function and is often disrupted in disease. Understanding how cells are organized in complex tissues has been advanced by technologies that analyze proteins, RNA, and DNA in their spatial contexts. However, existing spatial multiomics methods often rely on specialized equipment and reagents, limiting their accessibility. To address this, we developed SPatial assay for Accessible chromatin, Cell lineages, and gene Expression with sequencing (SPACE-seq), a technique that enables the simultaneous analysis of multiple molecular features-gene expression, chromatin accessibility, and mitochondrial DNA mutations-using a widely available spatial transcriptomics platform. SPACE-seq offers a unified molecular approach, providing a versatile solution for studying the multiomics landscape of complex tissues.

The spatial organization of cells within tissues is fundamental to their proper function in health and can go awry in disease. To understand the spatial organization of complex tissues, various technologies have been developed to examine spatial arrangement at the proteomic, transcriptomic, and epigenomic levels (1–4). While these innovative approaches have provided valuable insights into diverse biological contexts, methods that simultaneously capture multiple types of data–known as multiomics–are particularly valuable because they capture multiple aspects of cell state (5–10). However, the use of spatial multiomics methods is often limited by the need for custom-made devices and reagents. In order to make spatial multiomics more accessible, and to ensure that these methods can benefit from the continually improving cost and resolution of spatial transcriptomics methods, we aimed to develop a unified molecular strategy to capture multiple spatial modalities using a commercially available spatial transcriptomics platform.

**Fig. 1.**
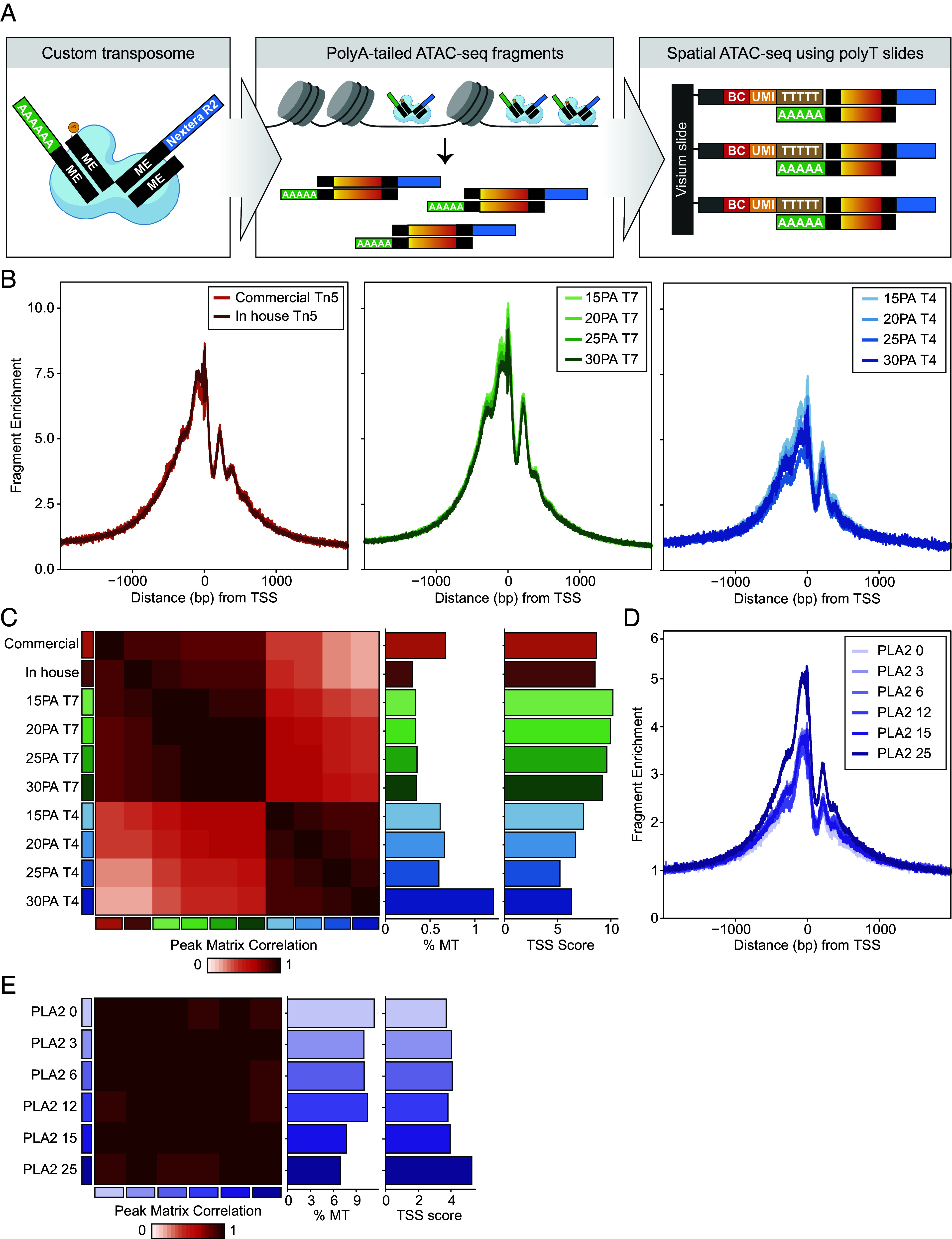
Optimization of transposome with polyA adaptors, barcode ligation, and permeabilization conditions for SPACE-seq. (*A*) Conceptual diagram illustrating the use of a polyA-tailed transposome to enable spatial epigenomics using slides designed for polyA capture chemistry. (*B*) TSS enrichment for bulk ATAC-seq samples performed using commercially available Tn5 versus Tn5 produced in-house using standard adapters (*Left*), T7 ligase and variable-length polyA adapters (*Center*), and T4 ligase and variable-length polyA adapters (*Right*). (*C*) Peak matrix correlation, percent mitochondrial reads, and TSS enrichment quantification for experiments with variable lengths of polyA adaptors and ligation conditions. (*D*) TSS enrichment of bulk ATAC-seq for mouse brain samples using standard adapters with varying duration (minutes) of phospholipase A2 (PLA2). (*E*) Peak matrix correlation, percent mitochondrial reads, and TSS enrichment quantification for PLA2 permeabilization optimization experiment.

Here, we develop SPatial assay for Accessible chromatin, Cell lineages, and gene Expression with sequencing (SPACE-seq), an unbiased and high-throughput spatial method that interrogates chromatin accessibility, mitochondrial mutations, and gene expression using the commercially available 10× Genomics Visium CytAssist platform (Fig. 1*A*). SPACE-seq adapts the assay for transposase-accessible chromatin using sequencing (ATAC-seq) to be compatible with the polyadenine (polyA) capture chemistry common in solid-phase spatial transcriptomics workflows, enabling one unified chemistry to investigate the spatial epigenomic and transcriptomic profiles of complex tissues.

ATAC-seq utilizes the hyperactive Tn5 transposase enzyme to simultaneously fragment DNA and insert custom adaptor sequences at the cut sites, which serve as PCR handles for amplification (11, 12). Commercially available Tn5 can be loaded with adaptor sequences of the user’s design. We hypothesized that by loading Tn5 with adaptors containing a 3′ polyA overhang, we could generate polyA-tailed chromatin fragments. These chromatin fragments could then be captured using standard spatial transcriptomics reagents, which typically bind polyA sequences (Fig. 1*A*).

To achieve this, we focused on three key steps: 1) developing the polyA-loaded transposome, 2) identifying ligation conditions that enable the attachment of spatial barcodes to the chromatin fragments, and 3) optimizing tissue permeabilization conditions to allow Tn5 access to nuclear DNA without compromising tissue integrity, as well as facilitating the release of chromatin fragments for capture by the spatial slide. We systematically tested all three parameters using bulk ATAC-seq (Fig. 1 *B*–*E*). We found that 15 base pair (bp) polyA adaptors provided higher enrichment at the transcription start site (TSS) and that T7 DNA ligase consistently yielded superior data quality compared to T4 DNA ligase (Fig. 1*B*). Moreover, combining 15 bp polyA adaptors with T7 DNA ligase yielded ATAC-seq data highly correlated with results from the standard ATAC-seq protocol using a Tn5 transposome with traditional adaptors (Fig. 1*C*). To enhance Tn5 access to nuclear DNA, we explored the use of phospholipase A2 (PLA2) to disrupt phospholipids in the cell membrane, hypothesizing that this might facilitate entry of the Tn5 transposome into the nucleus. The introduction of PLA2 improved the quality of bulk ATAC-seq data obtained from mouse brain samples (Fig. 1 *D* and *E*). Based on these findings, we proceeded with the use of 15 bp polyA adapters, T7 DNA ligase, and PLA2 in the development of SPACE-seq.

**Fig. 2.**
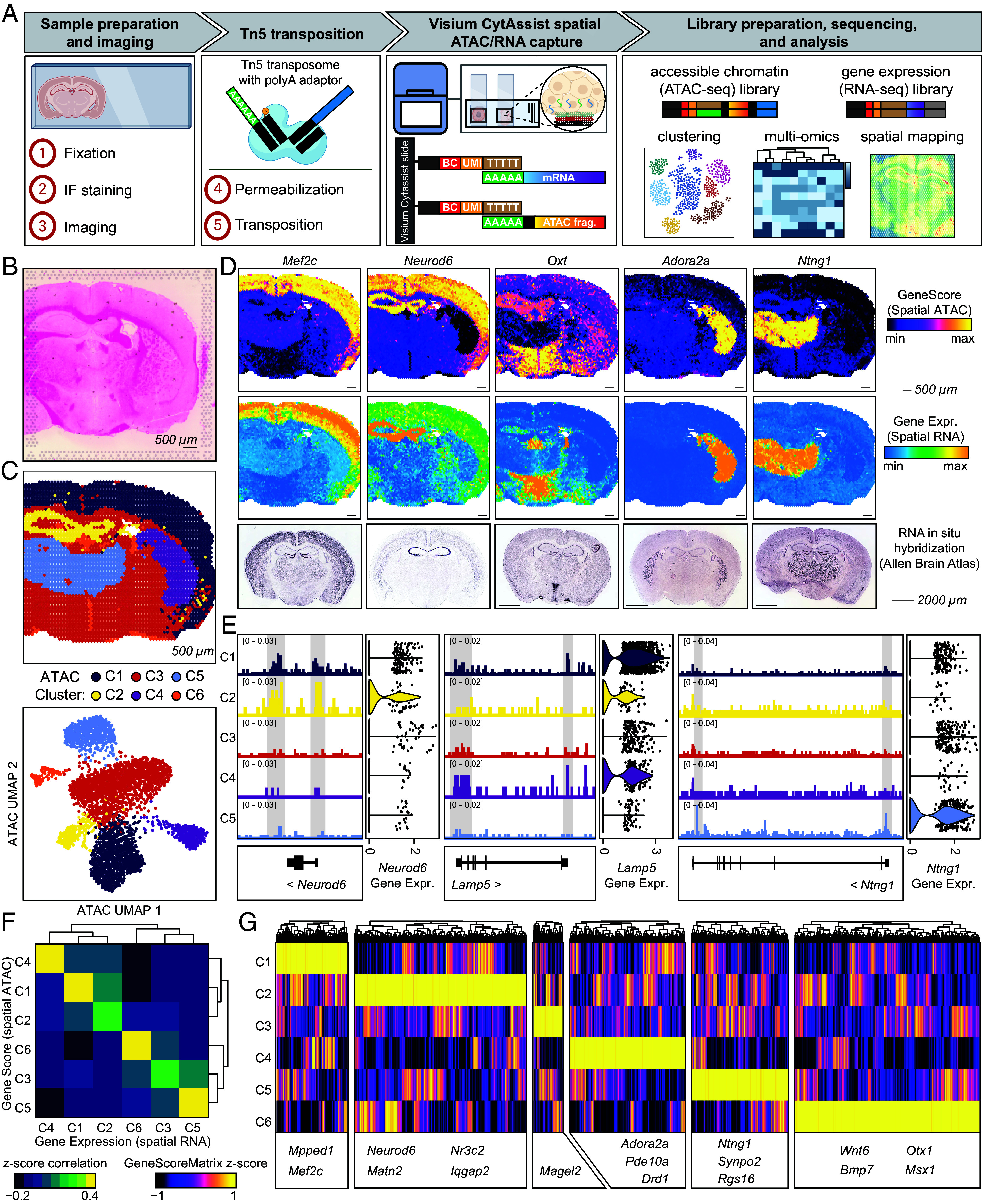
SPACE-seq enables simultaneous spatial profiling of accessible chromatin and gene expression using standard whole transcriptome reagents. (*A*) Schematic illustration of the SPACE-seq method. (*B*) Eosin-stained image of the mouse brain section obtained using the Visium CytAssist instrument. (*C*) Spatial ATAC-seq clusters visualized in their spatial context (*Top*) and after dimensionality reduction (*Bottom*). (*D*) Visualization of selected marker genes visualized in their spatial context and quantified using GeneScore (spatial ATAC-seq; *Top*), Gene Expression (spatial RNA-seq; *Center*), or RNA in situ hybridization (image credit: the Allen Brain Atlas; *Bottom*). (*E*) Genome coverage tracks from spatial ATAC-seq and expression levels from spatial RNA-seq for select marker genes, divided by cluster. (*F*) Correlation analysis between spatial RNA-seq GeneExpression and spatial ATAC-seq GeneScore for all genes significant in at least one pairwise comparison between clusters (*P* ≤ 0.05 and |log_2_ F.C.| ≥ 1 in either GeneScore or GeneExpression; n = 8,384 genes). (*G*) Heatmap displaying all differential genes significant in at least one pairwise comparison between clusters, quantified using GeneScore (*P* ≤ 0.05 and |log_2_ F.C.| ≥ 1; spatial ATAC-seq). Key marker genes are highlighted.

After optimizing key parameters using bulk ATAC-seq, we investigated whether our polyA-tailed ATAC fragments and polyadenylated mRNAs could be effectively captured using standard spatial transcriptomics reagents. We selected the spatially barcoded Visium slides from 10× Genomics due to their widespread availability and popularity (*SI Appendix*, Fig. S1). Using mouse brain tissue sections, we found that increasing the amount of Tn5 transposome was critical for enhancing fragment yield (*SI Appendix*, Fig. S1 *A* and *B*). We next compared the Visium v1 workflow with the Visium v2 CytAssist-enabled workflow and incorporated mRNA capture. The Visium v2 CytAssist-enabled SPACE-seq workflow resulted in a significantly larger median number of ATAC-seq fragments (3,810 Unique Molecular Identifiers (UMIs)) compared to the Visium v1 workflow (1,912 UMIs). In contrast, gene expression (GEX) UMIs were slightly lower in the Visium v2 workflow (2,473 UMIs) compared to the Visium v1 (4,254 UMIs) under comparable sequencing depth (*SI Appendix*, Fig. S1 *B*–*D*), indicating that CytAssist instrument may enhance the ATAC-seq/mRNA capture ratio, with the underlying mechanisms remain unclear. Notably, the SPACE-seq protocol can be effectively performed without the CytAssist instrument, but the quality of downstream analysis is suboptimal in this scenario. Therefore, we prioritized the Visium v2 CytAssist-enabled SPACE-seq workflow for further characterization (Fig. 2*A* and *SI Appendix*, Fig. S2).

In our mouse brain dataset, we identified 3,810 spots under tissue and performed additional quality control on the spatial ATAC and spatial RNA libraries (Fig. 2*B* and *SI Appendix*, Fig. S3 *A*–*C*). Unbiased dimensionality reduction and clustering of the spots demonstrated that both spatial ATAC and spatial RNA modalities independently recapitulated the known organization of the mouse brain. Specifically, cluster C1 corresponded to the cerebral cortex, cluster C2 to the hippocampus, cluster C4 to cerebral nuclei, and cluster C5 to the thalamus. (Fig. 2*C* and *SI Appendix*, Fig. S3*D*). This was further confirmed by visualizing both ATAC and RNA data for known marker genes such as *Mef2c*, *Neurod6,* and *Ntng1*, alongside reference in situ hybridization data from the Allen Brain Atlas (Fig. 2 *D* and *E* and *SI Appendix*, Figs. S3*E* and S4). We observed high concordance of the spatial RNA gene expression and spatial ATAC gene accessibility across clusters, as expected, and identified regulatory elements that may control the expression of these marker genes (Fig. 2 *F* and *G* and *SI Appendix*, Fig. S3*E*).

**Fig. 3.**
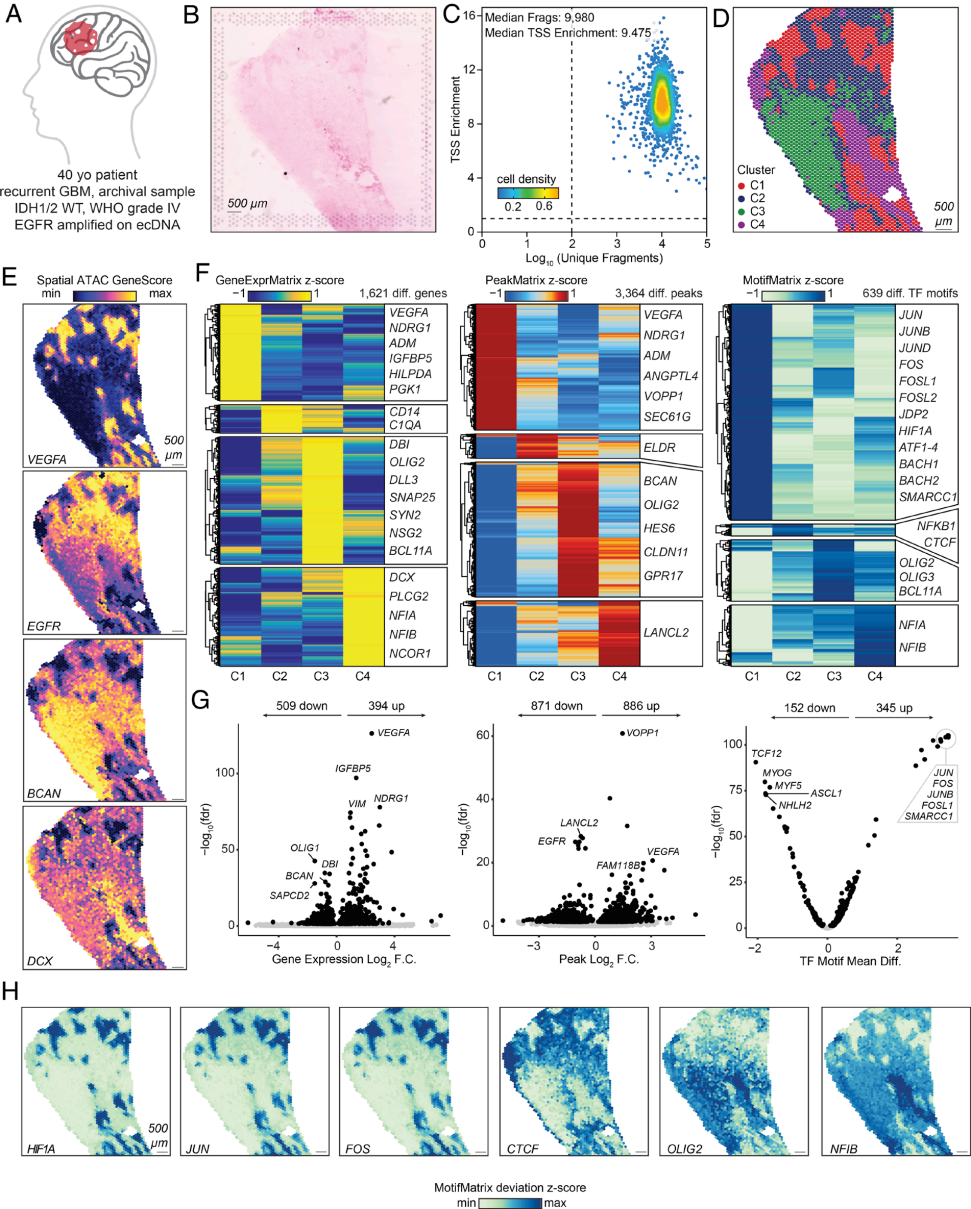
SPACE-seq reveals the spatial architecture and clonal landscape of an archival human glioblastoma sample. (*A*) Illustration and clinical characteristics of the human glioblastoma sample. (*B*) Eosin-stained image of the tumor section obtained using the Visium CytAssist instrument. (*C*) TSS enrichment and number of fragments per spot using spatial ATAC-seq. (*D*) Spatial distribution of each integrated cluster. (*E*) Visualization of selected marker genes visualized in their spatial context and quantified using GeneScore (spatial ATAC-seq). (*F*) Heatmaps displaying all differential items significant in at least one pairwise comparison between clusters for gene expression (*Left*), chromatin accessibility (*Center*), and transcription factor (TF) motifs (*Right*). Key marker genes are highlighted. (*G*) Comparison between C1 and C2-4 for gene expression (*Left*), chromatin accessibility (*Center*), and TF motifs (*Right*). (*H*) Selected TF motif deviations visualized in their spatial context.

**Fig. 4.**
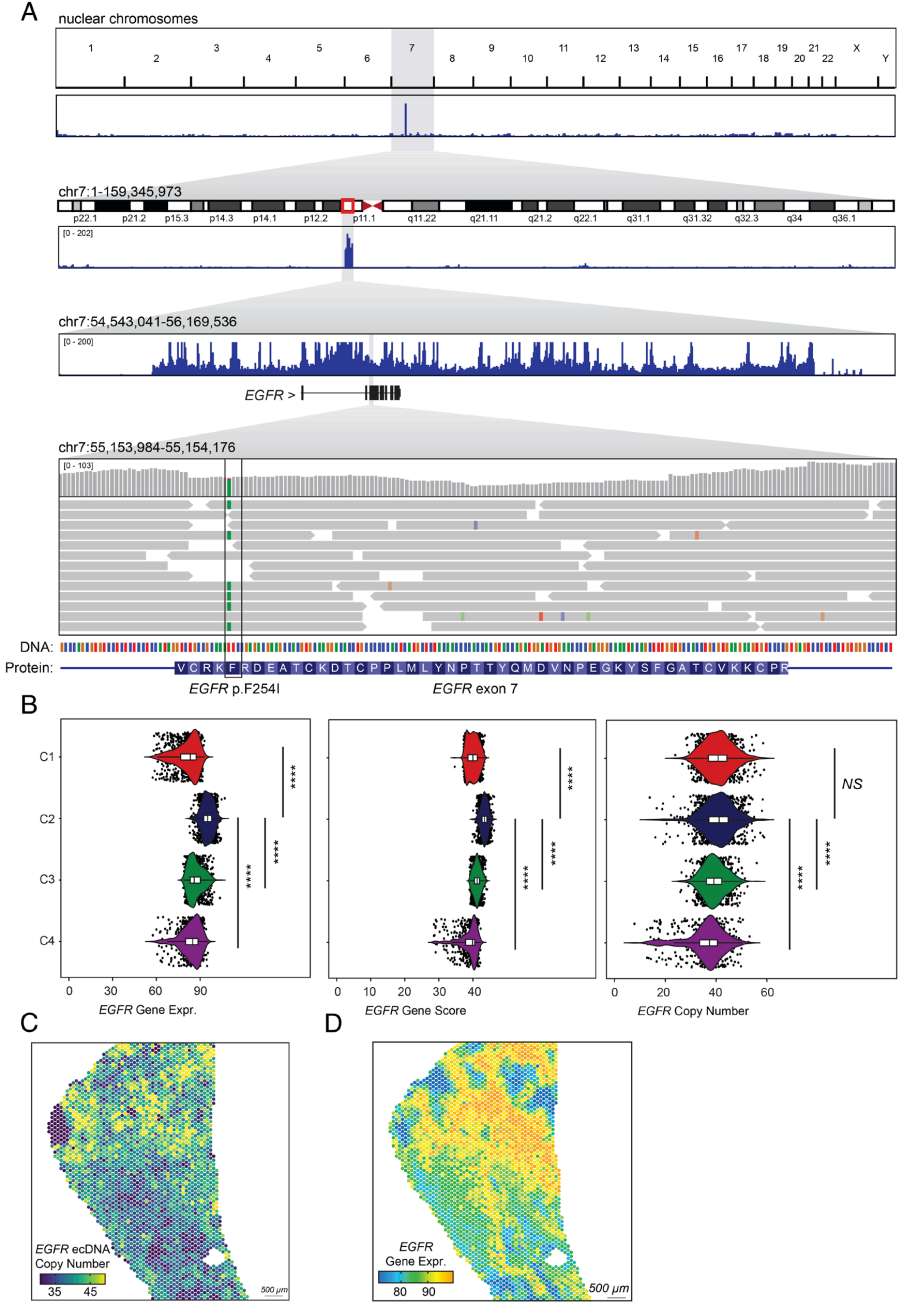
SPACE-seq provides insight into EGFR ecDNA in the human glioblastoma sample. (*A*) Spatial ATAC-seq genome coverage across all chromosomes, chromosome 7, the *EGFR*-amplified ecDNA, and *EGFR* exon 7 (in order *Top* to *Bottom*). In the *EGFR* exon 7 view, individual reads are shown in addition to genome coverage. (*B*) *EGFR* gene expression (*Left*), gene score from spatial ATAC-seq (*Center*), and copy number (*Right*) visualized for each cluster. Mann–Whitney test: ****P < 0.0001; NS indicates not significant. (*C*) Estimated *EGFR* ecDNA copy number per spot using spatial ATAC-seq. (*D*) Spatial distribution of *EGFR* gene expression.

Next, we applied SPACE-seq to an archived human glioblastoma sample (Fig. 3 *A* and *B*). Despite the sample being stored for several years, we obtained high-quality data for both spatial ATAC-seq and spatial RNA-seq (Fig. 3*C* and *SI Appendix*, Fig. S5*A*). We performed dimensionality reduction and clustering independently for each modality (*SI Appendix*, Fig. S5*B*), and then integrated the results to obtain unified cluster assignments incorporating both the ATAC and RNA data, identifying four distinct clusters (Fig. 3*D* and *SI Appendix*, Fig. S5*C*). Cluster C1 was the most distinct cluster and was marked by a mesenchymal-like hypoxia signature, including upregulation of genes such as *VEGFA*, *NDRG1*, *and ADM,* consistent with prior literature (Fig. 3*E* and *SI Appendix*, Fig. S6*A*) (13). Cluster C2 exhibited signatures of malignant mesenchymal-like cells, characterized by genes like *CLU*, *CD44,* and *C1R*, as well as macrophage markers *CD14* and *C1QA* (*SI Appendix*, Fig. S6*B*). Cluster C2 also showed the highest expression and accessibility for *EGFR*, which was amplified on extrachromosomal DNA (ecDNA) in this sample (Figs. 3*E* and 4). Cluster C3 displayed malignant oligodendrocyte progenitor-like cell signatures, with genes such as *BCAN*, *OLIG2,* and *TTYH1*, and astrocyte-like characteristics marked by *BCAN* and *S100B* (Fig. 3*E* and *SI Appendix*, Fig. S6*C*). Cluster C4 exhibited features of malignant neural progenitor-like cells, expressing genes like *DCX* and *RUNX1T1*, and showed enrichment of chromatin and transcriptional regulators such as *NFIA*, *NFIB*, and *PLCG2* (Fig. 3*E* and *SI Appendix*, Fig. S6*D*). These findings aligned well with previously published transcriptomic cellular phenotypes within glioblastoma (13–15). Moreover, our spatial epigenomic data also identified regulatory elements and transcription factors that may underlie the observed transcriptional phenotypes, such as *HIF1A*, a key responder to hypoxia, and activator protein 1 complex components, such as *JUN* and *FOS* family members in C1, *CTCF* in C2, *OLIG2/3* in C3, and *NFIA/B* in C4 (Fig. 3 *F*–*H*).

We then hypothesized that the spatial ATAC-seq reads mapping to the mitochondrial genome could be used to identify putative mitochondrial DNA (mtDNA) variants within the tumor (16–19) (*SI Appendix*, Fig. S7*A*). MtDNA is present at up to 1,000 copies per cell and has a mutation rate that is 100-fold greater than nuclear DNA, making it a powerful recorder of somatic cell lineages in adult humans (16, 17). Using the mgatk tool, we identified 12 putative mitochondrial variants with strand correlation >0.25 (*SI Appendix*, Fig. S7 *B* and *C*). All 12 variants exhibited heteroplasmy >50% and were generally prevalent throughout the glioblastoma tissue, suggesting a germline origin for these mutations. The minor alleles of these variants may represent somatic mitochondrial variation or the presence of nuclear-embedded mitochondrial DNA sequences (NUMTs) (20). While the young age of this donor likely precluded the identification of bona fide somatic variants, we proceeded to map the spatial locations of these putative variants to provide proof-of-concept for SPACE-seq’s ability to spatially localize mtDNA genotypes (*SI Appendix*, Fig. S7 *D*–*F*). These findings demonstrate that SPACE-seq is an effective tool for detecting mtDNA. In the mouse brain sample, many spots contained over 40% of spatial ATAC-seq reads mapped to mtDNA (*SI Appendix*, Fig. S3*B*), while in the glioblastoma sample, approximately 20% of spatial ATAC-seq reads mapped to mtDNA (*SI Appendix*, Fig. S7*A*). These results highlight the future potential of SPACE-seq to leverage mtDNA mutations for in situ lineage tracing.

Recent studies have demonstrated that single-cell ATAC-seq data can offer insights into additional aspects of tumor biology, such as ecDNA heterogeneity and somatic evolution (16, 18, 21, 22). We aimed to determine whether our spatial ATAC-seq data could serve similar purposes. First, we examined the spatial ATAC-seq signal from the extrachromosomally amplified region on chromosome 7p11 (Fig. 4*A*). As expected, this region exhibited high amplification, and we also identified a putative driver mutation within the *EGFR* gene (*EGFR* p.F254I; Fig. 4*A*). This mutation, located in the extracellular domain of EGFR, has been previously reported in other glioblastoma patients (23). Next, we estimated ecDNA copy number (CN) across the tumor and found that the *EGFR* ecDNA CN was enriched in mesenchymal-like clusters C1 and C2 (Fig. 4*B*). Interestingly, both gene accessibility and gene expression of *EGFR* were highest in cluster C2 and downregulated in hypoxic areas in cluster C1 (Figs. 3*E* and 4 *B*–*D*). These findings suggest that the tumor microenvironment, particularly hypoxia, may influence oncogene expression and chromatin accessibility of ecDNAs. These observations highlight the ability of SPACE-seq to investigate additional aspects of tumor biology—such as ecDNA CN and identification of putative driver mutations—that are not typically achievable with spatial transcriptomics.

In conclusion, SPACE-seq enables simultaneous capture of spatial ATAC-seq, RNA-seq, and mtDNA mutations using a standard whole transcriptome spatial platform. This approach allows for an unbiased dissection of multiple layers of cell state through a unified molecular strategy compatible with the widely used polyA capture chemistry in spatial transcriptomics. We envision that the method can be integrated and expanded to include additional data modalities. For instance, SPACE-seq could be adapted to incorporate spatial protein markers or spatial DNA methylation profiling. Moreover, our use of the polyA-loaded transposome may be beneficial for other techniques, such as Cleavage Under Targets and Tagmentation (CUT&TAG) (24). Given the compatibility of SPACE-seq with existing spatial transcriptomics reagents, and as the capabilities, resolution, and affordability of these platforms continue to improve, SPACE-seq may become an increasingly powerful approach for investigating the multiomics and clonal dynamics of complex tissues with exquisite spatial precision. Overall, we anticipate that SPACE-seq will accelerate progress in and democratize access to spatial multiomics.

## Methods

### Collection of Murine Tissue Samples.

All mouse work was conducted under Stanford’s approved animal protocol APLAC-14046. Wild-type C57BL/6 mice at 8 wk old were purchased from Jackson Laboratories and were then euthanized under Stanford University Administrative Panel on Laboratory Animal Care (APLAC) protocol No. 14046. The mouse brain tissues were harvested, snap-frozen in optimal cutting temperature (OCT) compound (Tissue-Tek, 4583) blocks in a dry ice-isopentane bath, kept on dry ice before sectioning, and stored at −80 °C.

### Collection of Tumor Samples from Patients with Glioblastoma.

*EGFR-*amplified glioblastomas were retrospectively identified in the Stanford University pathology database as approved by the Stanford University institutional review board (protocol 69198). Corresponding frozen glioblastoma tissue was obtained from the Stanford Cancer Institute Tissue Bank. The glioblastoma sample was then embedded in an OCT compound block in a dry-ice-isopentane bath, kept on dry ice before sectioning, and stored at −80 °C.

### Bulk ATAC-seq.

First, 50,000 viable cells were pelleted by centrifugation at 500× g for 5 min at 4 °C using a fixed-angle centrifuge. The supernatant was carefully aspirated without disturbing the cell pellet, first by removing the bulk down to 100 µL with a 1,000 µL pipette, followed by removing the remaining 100 µL with a 200 µL pipette. The cell pellet was then resuspended in 50 µL of cold ATAC Resuspension Buffer (RSB) containing 0.1% NP-40, 0.1% Tween-20, 0.01% digitonin, and 1× RNase A. The mixture was gently pipetted up and down three times and incubated on ice for 3 min.

Following incubation, 1 mL of cold ATAC RSB supplemented with 0.1% Tween-20 and 1× RNase A (but without NP-40 or digitonin) was added to wash out the lysis buffer. The tube was inverted three times to mix the contents. The nuclei were then pelleted by centrifugation at 500× g for 10 min at 4 °C. The supernatant was carefully removed using the two-step pipetting method to avoid disturbing the nuclei pellet.

The nuclei pellet was resuspended in 50 µL of transposition mixture by gently pipetting up and down six times. The transposition mix consisted of 25 µL of 2× TD buffer (20 mM Tris-HCl, 10 mM MgCl_2_, 20% dimethylformamide), 2.5 µL of transposome assembly, 16.5 µL of PBS, 0.5 µL of 1% digitonin, 0.5 µL of 10% Tween-20, 0.5 µL of 100× RNase A, and 4.5 µL of nuclease-free water. The transposition reaction was incubated at 37 °C for 30 min in a thermomixer.

For the primary amplification of transposed fragments, the transposed nuclei were resuspended in 50 µL of T7 ligation mix, which included 25 µL of 2× StickTogether Buffer, 2 µL of 25 µM SP-ATAC-Read1 primer, 2 µL of T7 ligase, and 21 µL of nuclease-free water. The mixture was incubated overnight at 25 °C. The reaction was then cleaned up using the Qiagen MinElute PCR Purification Kit (catalog number D4014). The DNA was eluted in 21 µL of elution buffer and stored at –20 °C until amplification. Typically, this elution yields approximately 20 µL of product, all of which was used in the subsequent PCR amplification. The bulk ATAC-seq library preparation was then prepared following OMNI-ATAC-seq protocol (12).

### Tn5 Transposome Assembly.

The assembly of Tn5 transpsome was performed as previously described (25). Initially, oligonucleotides (Tn5Me, Tn5MErev-15PA, Tn5ME-B, Tn5MErev) were prepared at a concentration of 100 μM each by resuspending them in water. Equal volumes of Tn5ME/Tn5MErev-15PA and Tn5ME-B/Tn5MErev were mixed separately in 200 μL PCR tubes to achieve a concentration of 50 μM each. These mixtures underwent denaturation at 95 °C for 5 min on a thermocycler and were then slowly cooled down by turning off the thermocycler. Subsequently, equal volumes of Tn5ME/Tn5MErev-15PA and Tn5ME-B/Tn5MErev were combined to achieve a concentration of 25 μM each. To make a stock of transposase adaptor, glycerol and oligo mixtures were then mixed in equal amounts to yield a concentration of 12.5 μM each. For each Visium reaction, Tn5 transposase assembly was as following: 7.5 μL transposase adaptors, 3.75 μL ddH_2_O and 3.75 μL Tn5 transposase (Diagenode, C01070010). The resulting solution was gently mixed and left at room temperature for 30 min to facilitate the annealing of oligos to Tn5.

### SPACE-seq.

Cryosections were prepared at a thickness of 10 µm using a cryostat (Epredia HM525 NX), placed onto glass slides, and kept on dry ice. A thermocycler adaptor (10× Genomics) was pre-equilibrated at 37 °C on a thermomixer, and tissue slides were incubated on the adaptor for 1 min.

10× Genomics Visium cassettes were assembled onto tissue slides. Each well was filled with 150 µL of 1% formaldehyde in Dulbecco’s Phosphate-Buffered Saline (DPBS) and incubated at room temperature for 10 min to fix the tissue. To quench the fixation, 150 µL of 1 M Tris-HCl was added to each well and incubated for 5 min. Next, the solution was aspirated, and 100 µL of DPBS was added, along with 1 U/µL RNase inhibitor (DPBS-I). After aspirating DPBS-I solution, 100 µL of blocking solution (2% bovine serum albumin, 0.01% Tween-20, 1 U/µL RNase inhibitor in DPBS) was applied to each well and incubated for 15 min at 4 °C. The blocking solution was then removed, and 100 µL of staining solution—comprising 0.5 µL DAPI and 0.5 µL wheat germ agglutinin antibody—was added to each well and incubated at 4 °C for 15 min. Each well was then washed once with 200 µL of DPBS-I. For imaging, 85% Glycerol in DBPS-I was added to slides, and coverslips were applied. After imaging, coverslips were removed by immersing slides in DPBS solution within a 50 mL tube, and tissues were rehydrated with DPBS.

Following rehydration, 100 µL of DPBS-I was added to each well. Subsequently, 100 µL of 0.1 U/µL porcine PLA2 (P6534, Sigma-Aldrich) with 1 U/µL RNase inhibitor was applied, and the slides were gently shaken at 300 rpm for 6 min. The slides were washed with DPBS-I and then treated with 100 µL of RSB buffer containing 0.1% NP40, 0.1% Tween-20, 0.01% Digitonin, and 1 U/µL RNase inhibitor for 10 min at room temperature, and 100 µL of RSB-TI (with 0.1% Tween-20 and 1 U/µL RNase inhibitor) was then added to each well for 5 min.

After aspiration, 100 µL of transposition mix containing 50 µL 2× TD buffer, 15 µL transposome assembly, 33 µL DPBS, 1 µL 1% digitonin, 1 µL 10% Tween-20, and 2.5 µL RNase inhibitor was added to each well. The slides were then incubated at 37 °C for 60 min with gentle shaking at 300 rpm. To halt the transposition reaction, 10 µL of 500 mM ethylenediaminetetraacetic acid (EDTA) (E0375, Teknova) was added to each well, and the slides were incubated at 37 °C for an additional 10 min. Each well was then washed with DPBS-I and added with 100 µL of a 5× saponin solution (50 µL 10× saponin, 47.5 µL DPBS, and 2.5 µL RNase inhibitors) and was incubated at 4 °C for 10 min.

After an additional washing step with DPBS-I, 150 µL of 10% Eosin was applied to the slides for a 1-min incubation at room temperature to stain the tissues. The slides were washed once with DPBS-I and, when completely dehydrated, placed on the tissue slide stage of the Cytassist machine. A permeabilizing mixture was prepared containing 50 µL of RNase buffer (PN-2000551, 10× Genomics), 7.5 µL of ddH2O, 7.5 µL of 10% sodium dodecyl sulfate (SDS) (15553027, ThermoFisher Scientific), and 10 µL of tissue removal enzyme (PN-300387, 10× Genomics). The Visium Cytassist slide was placed on the Visium slide stage and 25 µL of the permeabilizing mixture was added to each spacer well on the visium cytassist slide. The lid of the Cytassist machine was closed, and then the assembly was incubated at 37 °C for 30 min.

Following a DPBS-I wash, 100 µL of T7 ligation mixture (50 µL of 2× sticktogether buffer, 5 µL of T7 ligase, 2.5 µL of RNase inhibitor and 42.5 µL of ddH_2_O) was applied to each well and incubated at 25 °C for 2 h. After ligation, each well was aspirated and 75 µL of reverse transcription mixture (10× Genomics) was added. The slides were incubated at 53 °C for 45 min. The solution was then removed, and 150 µL of 1× NEBNext polymerase mixture was added; the slides were incubated at 72 °C for 15 min.

To reverse cross-link the tissues, each well was treated with 200 µL of a solution containing 50 mM Tris-HCl, 1 mM EDTA, 1% SDS, 200 mM NaCl, and 0.8 mg/mL Proteinase K. The slides were incubated at 58 °C for 1 h. After washing with DPBS and elution buffer, the ATAC-seq fragments were eluted by treating the wells with 35 µL of 0.08 N NaOH for 10 min. The eluted ATAC-seq fragments were neutralized with 5 µL of 1 M Tris-HCl and transferred to PCR tubes. Each well was then washed with DPBS and elution buffer.

For second strand synthesis (SSS), an SSS solution (10× Genomics) was added to each well and incubated at 65 °C for 15 min. Following this, the slides were washed with elution buffer (19086, Qiagen), and the cDNA was eluted with 35 µL of 0.08 N NaOH and neutralized with 5 µL of 1 M Tris-HCl.

### Library Preparation and Sequencing.

Spatially barcoded single-stranded cDNA fragments (GEX) were then prepared following the 10× Genomics Gene Expression protocol, starting with step 4 cDNA amplification and Visium Spatial Gene Expression Library Construction. Further, the spatial barcoded ATAC fragments were amplified using Truseq partial read 1 and Nextera partial read 2 with NEBNext master mix. The amplified products were purified using 0.9× SPRIselect beads (B23318, Beckman Coulter) and the primer dimers (~130 bp) were removed by gel extraction using E-gel EX 2% agarose. The gel-extracted products were then amplified using i7 and i5 indexed primer using NEBNext master mix. The final indexed libraries were cleaned up using 0.8× SPRIselect beads. The average size of libraries was measured by high sensitivity D5000 ScreenTape (5067-5592, Agilent) using tapestation and the concentration of libraries was measured by NEBNext library quantification kit for illumina (E7630L, NEB). Pooled libraries were sequenced on Illumina Nextseq 550 instrument. We sequenced 29 bases for read1, 10 bases for i7 and i5 index, and 123 bases for read2. In general, we recommend a minimum of 50,000 read pairs per spot for both ATAC-seq and RNA-seq signals, as suggested by 10× Genomics, to ensure adequate signal representation. For optimal results using SPACE-seq, we recommend selecting tissues with an RNA integrity number greater than 7, as suggested by 10× Genomics, given the positive correlation we observed between RNA integrity and the quality of ATAC-seq results.

### Spatial RNA-seq Analysis.

Spatial RNA files were processed using the spaceranger count pipeline from 10× Genomics (version 2.1.1., available on the 10× Genomics website). Given that our protocol uses the Visium Cytassist slide but not a probe set, we modified the spaceranger count pipeline to process our files using the polyA-capture settings typically used for the Visium V1 slides, but with the spatial barcodes and coordinates of a Visium Cytassist slide. To accomplish this, we used the spaceranger count settings for a Visium V1 slide, using a brightfield image and parameter “--unknown-slide visium-1. Second, we redirected spaceranger to use the spatial barcodes of the Visium Cytassist slide instead of the Visium V1 spatial barcodes by deleting the three files “visium-v1_coordinates.txt,” “visium-v1.gal,” and “visium-v1.txt” and renaming the corresponding “visium-v4” files to take their place. These files can be located and modified within the spaceranger installation under, for example, “spaceranger-2.1.1/lib/python/cellranger/barcodes.” Mouse samples were analyzed using the “refdata-gex-mm10-2020-A” reference genome from 10×, and human samples were analyzed using “refdata-gex-GRCh38-2020-A.”

After initial mapping, barcode correction, and quality control were performed by spaceranger, the 10× filtered counts matrix was loaded into Seurat v5 for downstream analysis. Normalization, dimensionality reduction, and clustering were performed in Seurat. For consistency with spatial ATAC-seq analysis, after initial quality control using Seurat, gene expression data were loaded into an ArchR GeneExpression matrix, and dimensionality reduction, clustering, and imputation were performed using ArchR. All barcodes were retained for analysis; no additional filtering was performed based on mitochondrial reads, nCounts, or nFeatures. Visualizations were created in R using the package ggplot2.

### Spatial ATAC-seq Analysis.

To ensure consistency between the RNA and ATAC barcode and UMI correction workflows and spatial mapping, spatial ATAC data were first processed using spaceranger with identical settings as described above for spatial RNA data. A table was created from the 10× spaceranger bam file mapping each read name to the associated cell barcode and UMI (found in the CB and UB bam tags). In parallel, the spatial ATAC files were processed using a more conventional ATAC-seq pipeline. Because read1 was used to capture the UMI and cell barcode, only read2 was used for ATAC-seq analysis. Reads were trimmed to a maximum length of 75 bp and adapter sequences were removed using “fastp.” Hisat2 was used to align fastq files to the mm10 or hg38 reference genome using parameters “--no-spliced-alignment --very-sensitive -X 2000.” The CB and UB tags were added to the hisat2 ATAC-seq alignments using the read name table created from spaceranger as well as a custom python script utilizing the “simplesam” package. Duplicates were removed from the bam file using the “dedup” utility from the “umi_tools” package based on both the cell barcode and umi tags. Finally, a synthetic paired end fragments file was created from the single-end alignments for compatibility with ArchR.

ArchR was used for downstream spatial ATAC-seq analysis. The “createArrowFiles” step was modified for spatial ATAC-seq by using the permissive parameters “minFragSize=-1,” “maxFragSize=10**10,” “minFrags=100,” and “minTSS=1” to retain nearly all barcodes for downstream analysis. Second, a “tileSize” of 5 kb was used for the ArchR TileMatrix. After creation of Arrow files, data preprocessing, dimensionality reduction, clustering, and imputation were performed in ArchR. Barcode spatial positions were loaded into the ArchR metadata to facilitate visualization. As described above for the spatial RNA data, visualizations were created in R using ggplot2.

### ecDNA CN Estimation.

We estimated ecDNA CN from background ATAC-seq signals as previously described and validated (21, 22). Briefly, we measured read counts in 3-megabase windows sliding across the genome in 1-megabase increments. Regions with known mapping artifacts were excluded using the ENCODE hg38 blacklist. For each window, we calculated the number of insertions per base pair and compared it to 200 neighboring windows with similar GC content and then computed the mean log2 fold change (FC). Assuming a diploid genome, we determined the CN using the formula:

$$\text{CN}=2*\left[2^{\wedge }\log2\left(\text{FC}\right)\right].$$

To find a gene’s CN, we identified all windows overlapping that gene’s annotated sequence and calculated the mean CN of those windows.

### Analysis of mtDNA Mutations from Spatial ATAC-seq Data.

To identify putative mtDNA variants from our spatial ATAC-seq data, we first repeated the spatial ATAC-seq pipeline described above using a previously described reference genome where nuclear regions of hg38 that mtDNA may erroneously align to are removed, to enable uniform mapping of reads across the mitochondrial genome (18). The quality-filtered and deduplicated bam file was then passed to mgatk in tenx mode for variant calling (18). Pass filter variants were determined based on variants that had a strand correlation of at least 0.25. Variant heteroplasmy for each of these variants for each cell was then loaded into the original ArchR project metadata for visualization and analysis.

## Supplementary Material

Appendix 01 (PDF)

Dataset S01 (XLSX)

Dataset S02 (XLSX)

Dataset S03 (XLSX)

## Data Availability

Code to process SPACE-seq data is available at https://github.com/juliabelk/SPACE-seq (26). Data are available in the NCBI Gene Expression Omnibus under accession GSE279771 (27).
