# Supplementary material for: Unified molecular approach for spatial epigenome, transcriptome, and cell lineages
Source: Proc Natl Acad Sci U S A. 2025 Apr 18;122(16):e2424070122. doi: 10.1073/pnas.2424070122 (PMC12037033; doi:10.1073/pnas.2424070122)
Supplement: Supplementary file 1 — Appendix 01 (PDF) [file pnas.2424070122.sapp.pdf]

PNAS

Supporting Information for

Unified molecular approach for spatial epigenome, transcriptome, and cell lineages

Yung-Hsin Huang[1,*], Julia A. Belk[1,*], Ruochi Zhang[2,3,4], Natasha E. Weiser[1,5], Zachary Chiang[2,3,4], Matthew G. Jones[1], Paul S. Mischel[5,6], Jason D. Buenrostro[2,3,4], Howard Y. Chang[1,7,8#]

[1] Center for Personal Dynamic Regulomes and Program in Epithelial Biology, Department of Dermatology, Stanford University School of Medicine, Stanford, CA, USA.
[2] Broad Institute of MIT and Harvard, Cambridge, MA, USA.
[3] Department of Stem Cell and Regenerative Biology, Harvard University, Cambridge, MA, USA.
[4] Gene Regulation Observatory, Broad Institute of MIT and Harvard, Cambridge, MA, USA.
[5] Department of Pathology, Stanford University, Stanford, CA, USA.
[6] Sarafan Chem-H, Stanford University, Stanford, CA, USA.
[7] Howard Hughes Medical Institute, Stanford University, Stanford, CA, USA.
[8] Current address: Amgen Research, South San Francisco, CA, 94080

*These authors contributed equally.
# email: howchang@stanford.edu

This PDF file includes:

Figures S1 to S7
Supporting text

Other supporting materials for this manuscript include the following:

Datasets S1 to S3

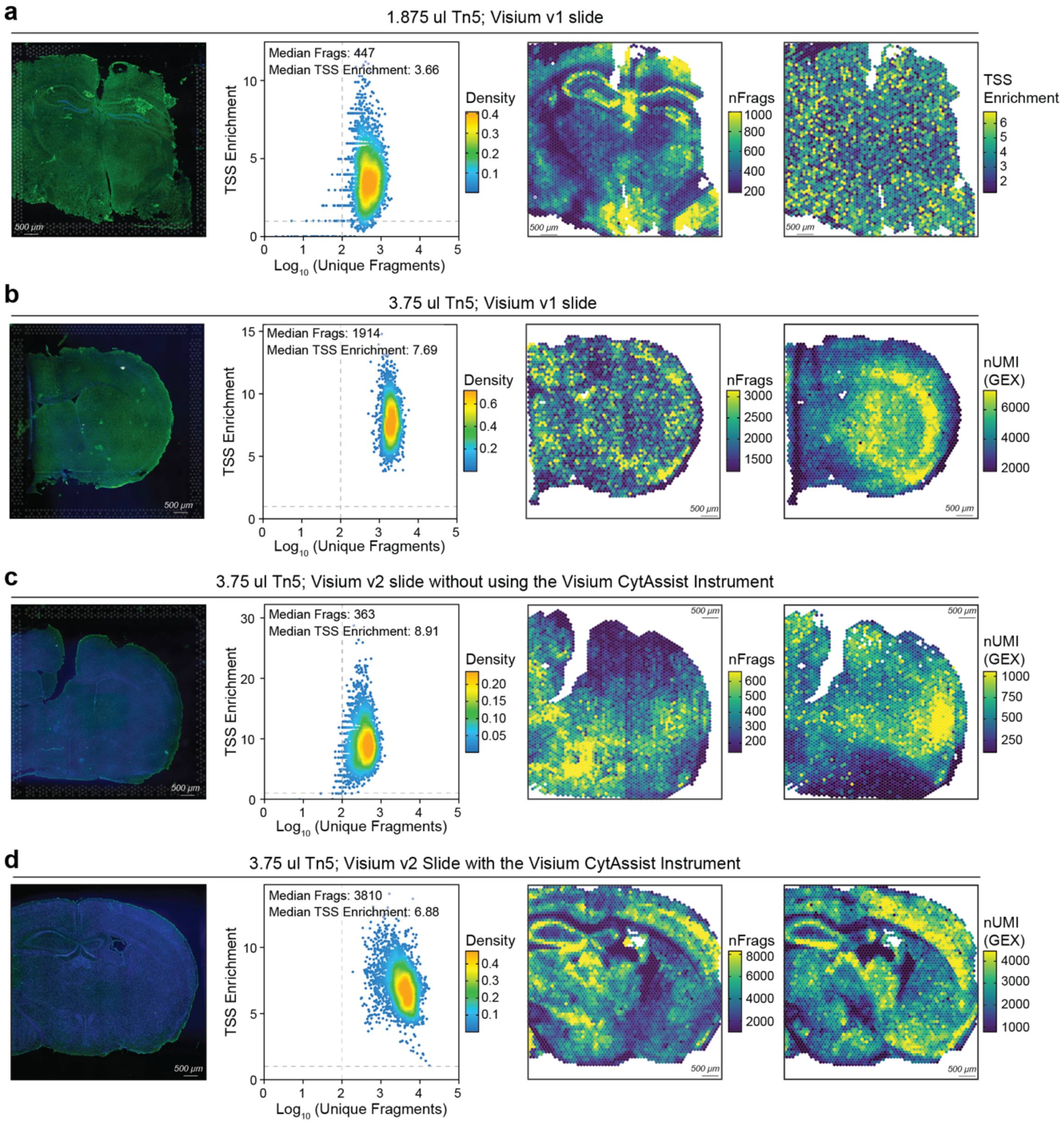

Fig. S1: Development of SPACE-seq using commercially available spatial transcriptomics reagents. Fluorescent image, number of fragments per spot, and TSS enrichment for spatial ATAC-seq using **(a)** the 10X Visium v1 slide and 1.875 uL Tn5. Fluorescent image, number of fragments per spot, and number of unique molecular identifiers (UMIs) of transcripts for SPACE-seq using **(b)** the 10X Visium v1 slide, **(c)** the 10X Visium v2 slide without using the Visium CytAssist instrument, or **(d)** the 10X Visium v2 slide with the Visium CytAssist instrument. (b-d) were performed using 3.75 uL Tn5.

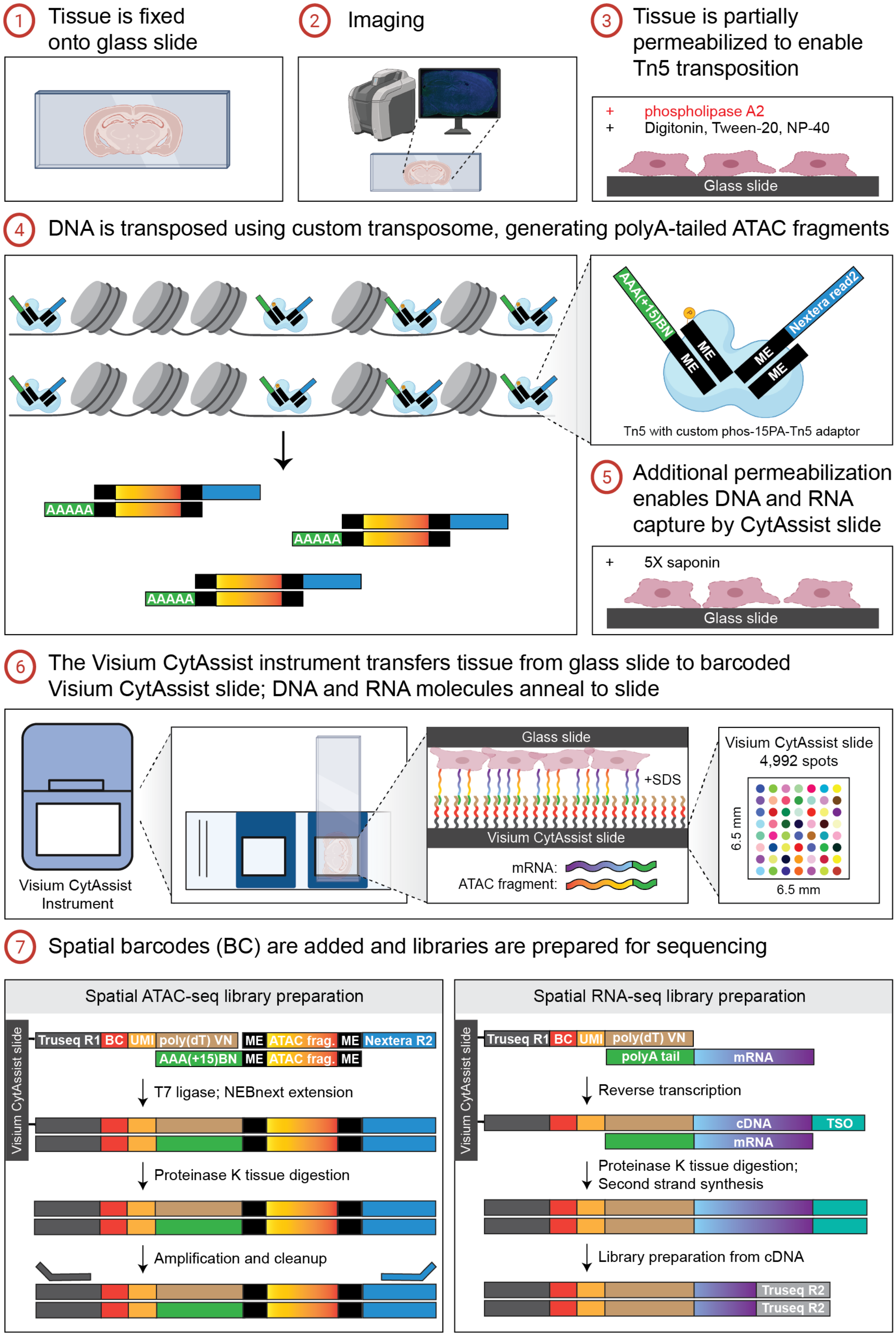

Fig. S2: Detailed schematic of the SPACE-seq method. (1) Tissues are fixed onto glass slides. **(2)** Tissue imaging is performed after immunofluorescence

staining. **(3)** Tissues are partially permeabilized by phospholipase A2, digitonin, tween-20 and NP-40. **(4)** Tissues undergo transposition using a custom transposome, generating polyA-tailed ATAC-seq fragments. **(5)** A 5X saponin solution is applied to further permeabilize the tissues. **(6)** The Visium CytAssist instrument enables efficient capture of ATAC-seq fragments and mRNAs. **(7)** Spatial barcodes are added to ATAC-seq fragments and cDNAs for subsequent library preparation.

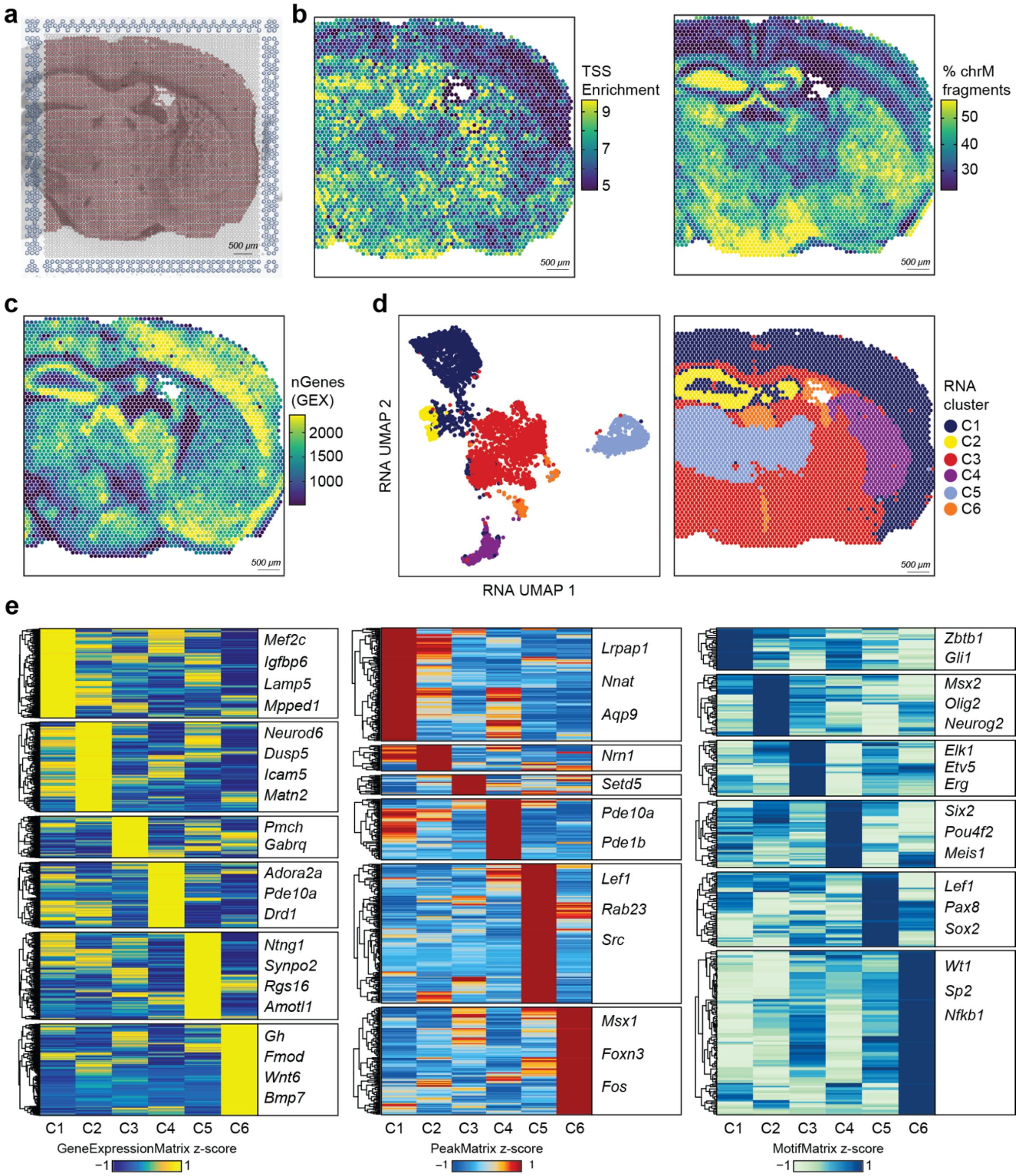

Fig. S3: Additional characterization of SPACE-seq performance on mouse brain. (a) Image of the tumor section obtained using the Visium CytAssist instrument. **(b)** TSS enrichment (left) and percentage of mitochondrial chromosome reads (right) per spot using spatial ATAC-seq. **(c)** Number of genes detected per spot using spatial RNA-seq. **(d)** Spatial RNA-seq clusters shown after dimensionality reduction (left) and in their spatial distribution (right). **(e)** Heatmaps displaying all differential items significant in at least one pairwise comparison between clusters for gene expression (left), chromatin accessibility (center) and transcription factor (TF) motifs (right). Key marker genes are highlighted.

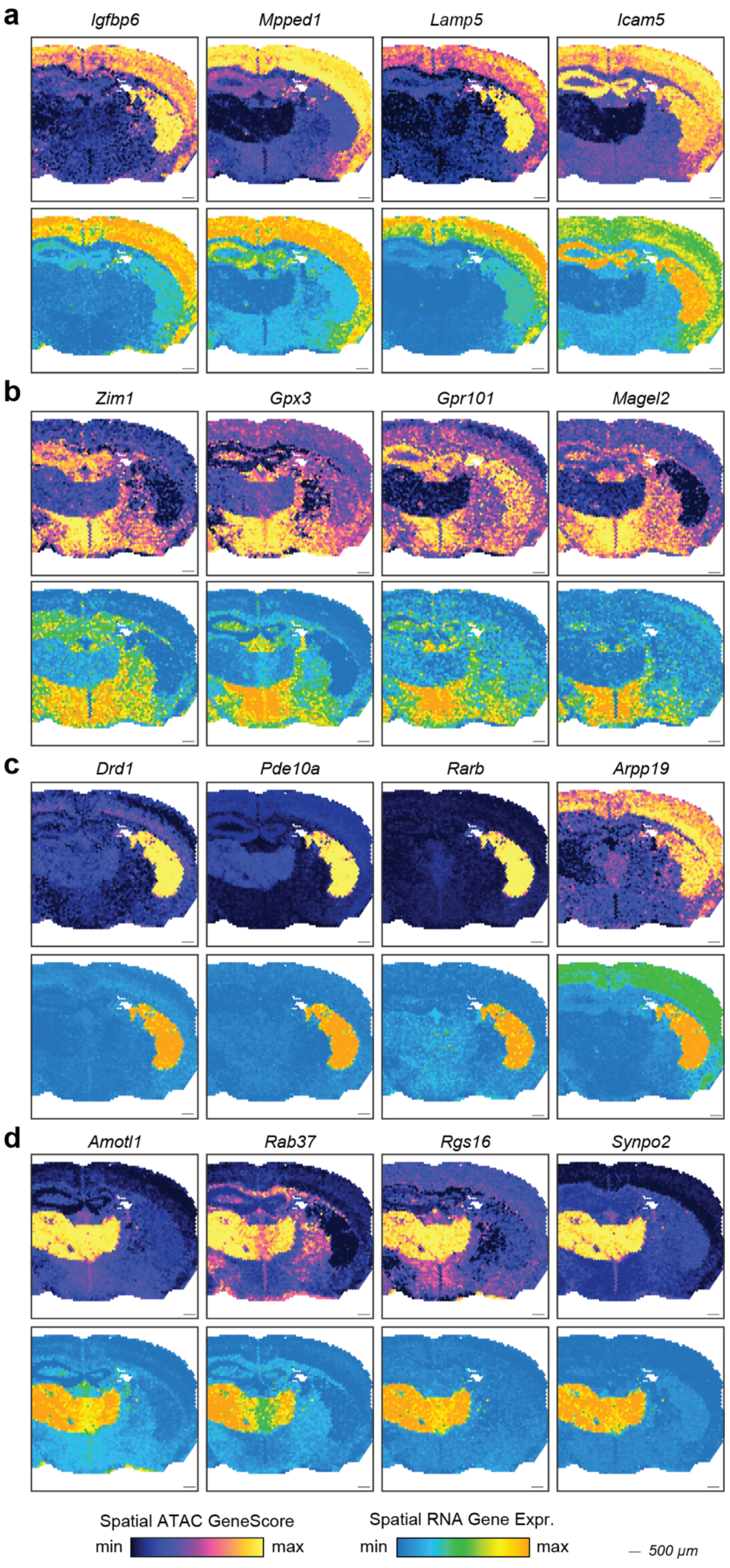
a
Igfbp6
Mpped1
Lamp5
Icam5
b
Zim1
Gpx3
Gpr101
Magel2
c
Drd1
Pde10a
Rarb
Arpp19
d
Amotl1
Rab37
Rgs16
Synpo2
Spatial ATAC GeneScore
min
max
Spatial RNA Gene Expr.
min
max
500 μm

Fig. S4: Additional spatial distributions of marker gene expression and accessibility in the mouse brain. (a-d) Selected marker genes visualized in their spatial context and quantified using GeneScore (spatial ATAC-seq; top) or Gene Expression (spatial RNA-seq; bottom).

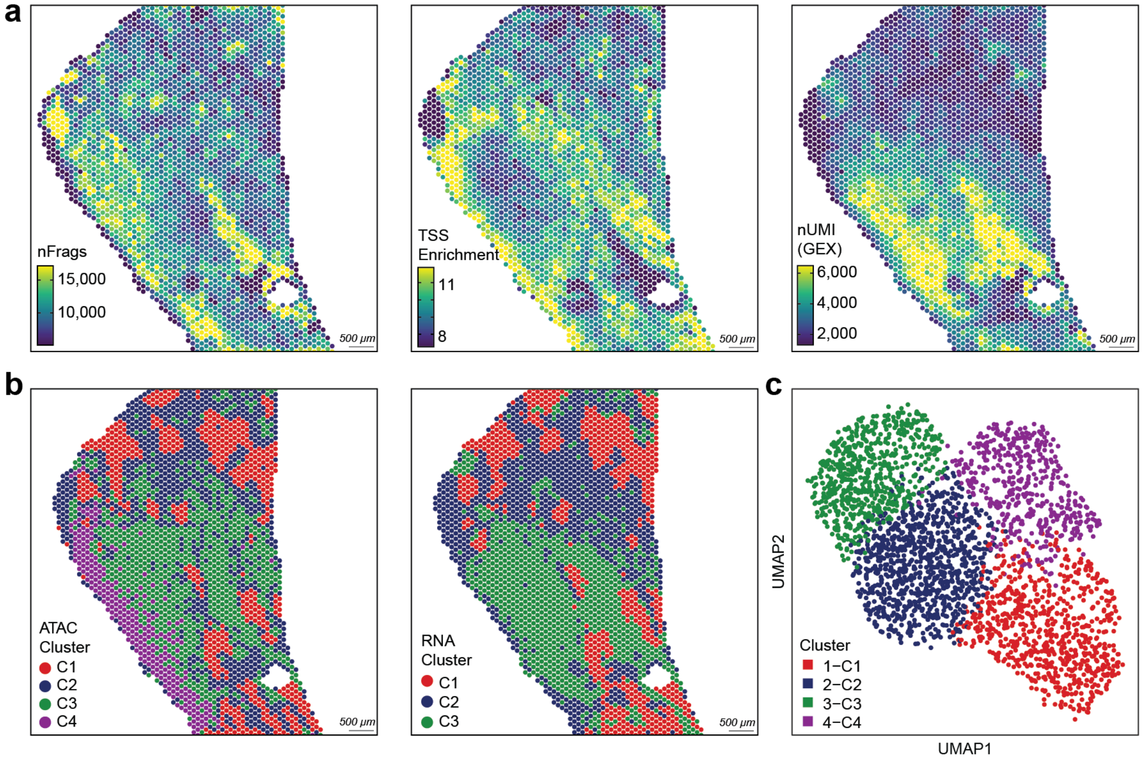

Fig. S5: Additional characterization of SPACE-seq performance on a human glioblastoma sample. (a) Number of fragments (left), TSS enrichment (center) per spot for spatial ATAC-seq, and number of UMIs (right) for spatial RNA-seq per spot. **(b)** Spatial ATAC-seq (left) or spatial RNA-seq (right) clusters shown in their spatial context (left). **(c)** Integrated clusters after dimensionality reduction.

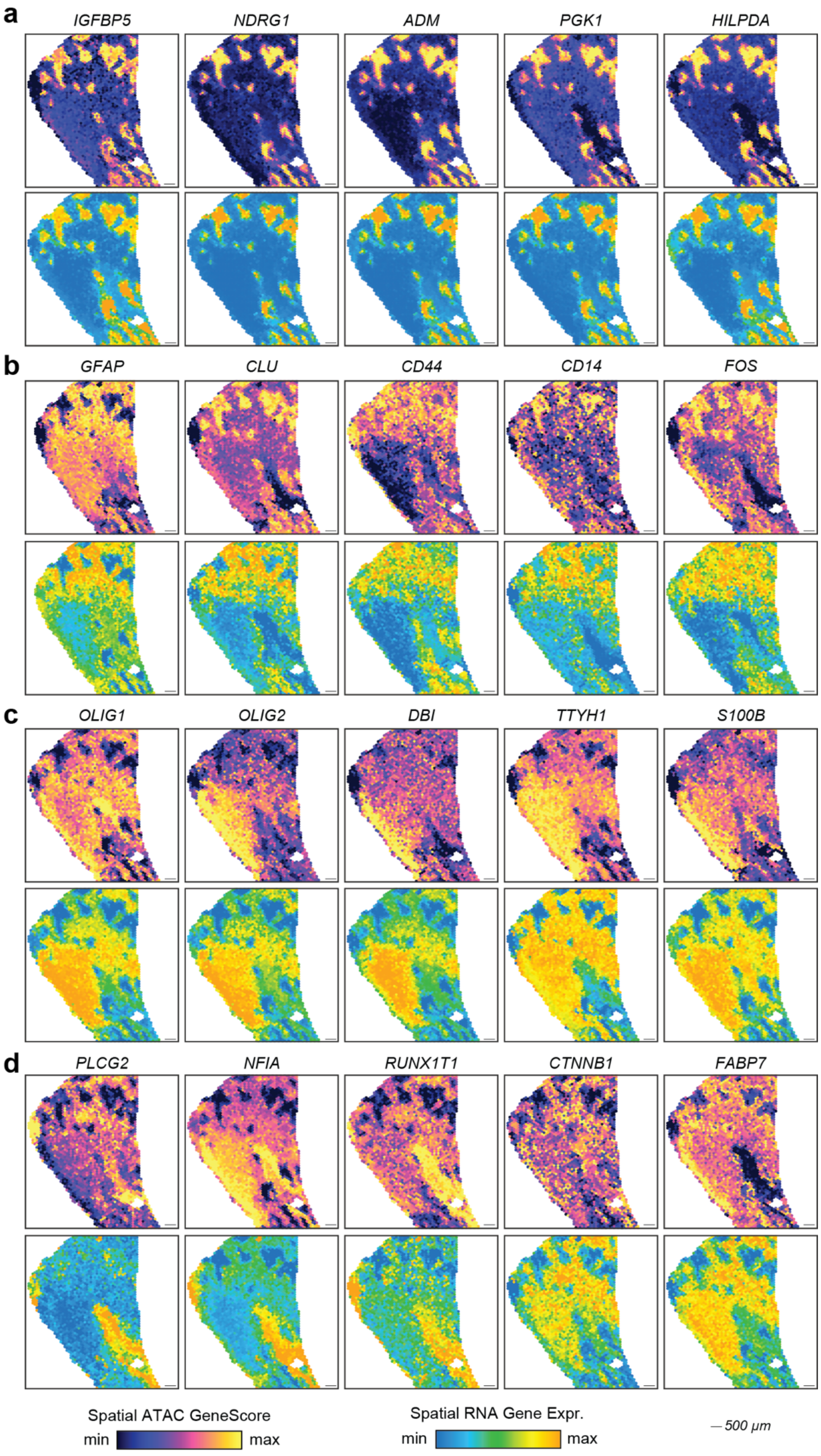
a
IGFBP5
NDRG1
ADM
PGK1
HILPDA
b
GFAP
CLU
CD44
CD14
FOS
c
OLIG1
OLIG2
DBI
TTYH1
S100B
d
PLCG2
NFIA
RUNX1T1
CTNNB1
FABP7
Spatial ATAC GeneScore
min
max
Spatial RNA Gene Expr.
min
max
500 μm

Fig. S6: Additional spatial distributions of marker gene expression and accessibility in a human glioblastoma sample. (a-d) Selected marker genes visualized in their spatial context and quantified using GeneScore (spatial ATAC-seq; top) or Gene Expression (spatial RNA-seq; bottom).

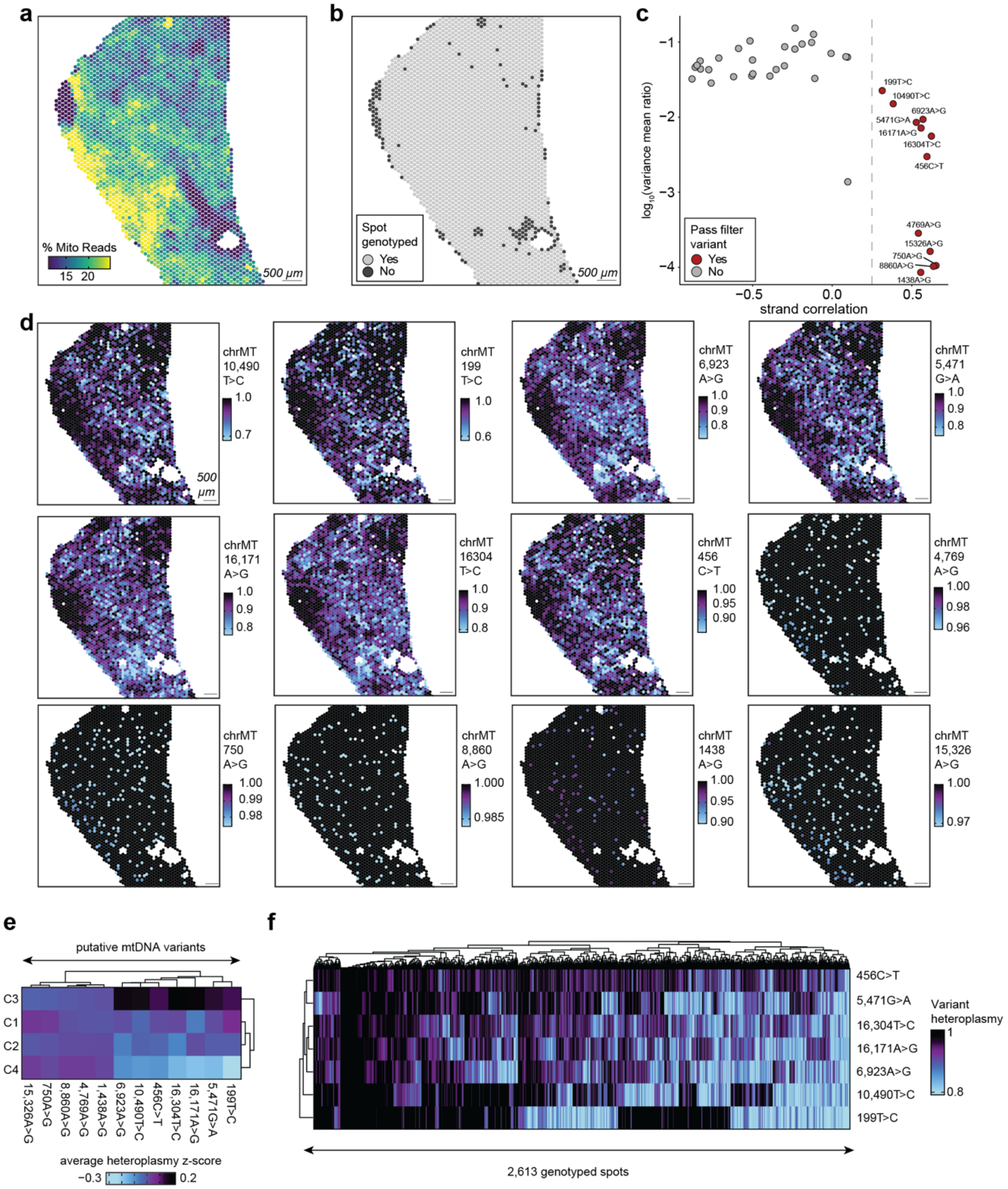

Fig. S7: SPACE-seq identifies putative mitochondrial DNA variants. (a) Percentage of mitochondrial reads per spot using spatial ATAC-seq. **(b)** Per-spot mitochondrial genotyping using spatial ATAC-seq. **(c)** Variance mean ration and strand concordance of each putative variant. **(d)** Spatial distribution of 12 mitochondrial DNA variants. **(e)** Quantification of the enrichment of each putative mitochondria DNA mutation in each cluster. **(f)** Heatmap showing variant heteroplasmy levels in individual spots for seven mitochondrial variants.

SPACE-seq protocol using 10X Visium v2 CytAssist-enabled slides

Tn5 transposome assembly

1. Oligonucleotides (oligo) (Tn5Me, Tn5MErev-15PA, Tn5ME-B, Tn5MErev) were resuspended in water to a final concentration of 100 μM each.
2. Equimolar amounts of Tn5ME+Tn5MErev-15PA and Tn5MErev+Tn5ME-B were mixed in separate 200 μl PCR tubes to 50 μM each.
3. These two tubes of oligo mixtures were denatured on a thermomixer for 5 min at 95 °C and cooled down slowly on the thermocycler by turning off the thermomixer.
4. Equimolar amounts of Tn5ME/Tn5MErev-15PA and Tn5MErev/Tn5ME- B were mixed to 25 μM each.
5. The D-Tn5 transposase was assembled with the following components: 7.5 ul transposase adaptors (final concentration of each double-stranded oligo was 12.5 μM each), 3.75 ul ddH$_2$O and 3.75 ul Tn5 (Diagenode, C01070010).
6. The reagents were mixed thoroughly but gently, and the solution was left on the bench at room temperature for 1 hr to allow annealing of oligos to Tn5.

SPACE-seq

1. Section tissue at 10 μm using cryostat on the day of experiment, place tissue sections on glass slides and keep glass slide with tissue section on dry ice.
2. A thermocycler adaptor is pre-equilibrated at 37 °C and tissue slide are placed on thermocycler adaptor for 1 minute.
3. 10X Genomics Visium CytAssist cassette are assembled onto tissue slides.
4. Completely immerse wells with 150 ul 1% formaldehyde in DPBS and incubate 10 minutes at room temperature.
5. Add 150 ul 1M Tris-HCl to wells and incubate for 5 minutes at room temperature.
6. Aspirate solution and add 100 ul DPBS with 1 U/ul RNase inhibitor (NEB, M0541L) (DPBS-I).
7. Aspirate solution and add 100 ul blocking solution (2% BSA, 0.01% Tween-20, 1U/ul RNase inhibitor in DPBS) for 15 minutes at 4 °C.
8. Aspirate solution and add 100 ul staining solution (0.5 ul DAPI and 0.5 ul WGA antibody in blocking solution) for 15 minutes at 4 °C.
9. Remove immunofluorescence staining solution and wash once with 200 ul DPBS-I.
10. Add 85% Glycerol with 1 U/ul RNase inhibitor in 1X DPBS to the slide, apply a coverslip slide, and apply coverslip and proceed to imaging.

11. After imaging, remove coverslip by dipping slide to DPBS solution in 50 ml tube.
12. Rehydrate with DPBS and remove the solution.
13. Add 100 ul 0.1 U pPLA2 (Sigma-Aldrich, P6534) in DPBS-I and incubate for 6 minutes at 37 °C with 300 rpm shaking.
14. Wash with 100 ul DPBS-I.
15. Add 100 ul ATAC-Resuspension Buffer (ATAC-RSB; 10 mM Tris-HCl, 10 mM NaCl, 3 mM $MgCl_2$) containing 0.1% NP40, 0.1% Tween-20, 0.01% Digitonin, and 1U/ul RNase inhibitor.
16. Incubate for 10 minutes at room temperature.
17. Wash out lysis with 100 ul of ATAC-RSB containing 0.1% Tween-20, and 1U/ul RNase inhibitor but NO NP40 or digitonin for 5 minutes at room temperature.
18. Aspirate ATAC-RSB solution completely.
19. Add 102.5 ul Transposition mix = (50 ul 2x TD buffer [20 mM Tris-HCl, 10 mM $MgCl_2$, 20% Dimethyl Formamide], 15 ul transposome assembly, 33 ul DPBS, 1 ul 1% digitonin, 1 ul 10% Tween-20, 2.5 ul RNase inhibitor)
20. Incubate reaction at 37 °C for 60 minutes with 300 rpm shaking.
21. Add 10 ul 500 mM EDTA and incubate 37 °C for 10 minutes.
22. Wash with 100 ul DPBS-I.
23. Remove solution and add 100 ul 5X Saponin solution (50 ul 10X Saponin, 47.5 ul DPBS and 2.5 ul RNase inhibitors in DPBS) at 4 °C for 10 minutes.
24. Wash with 100 ul DPBS-I.
25. Add 150 ul 10% Eosin to slide and incubate for 1 minute at room temperature.
26. Rinse with 250 ul DPBS-I.
27. Add 25 ul Rnase Buffer mixture from 75 ul mastermix (50 ul RNase Buffer B [10X Genomics, PN-2000551], 7.5 ul ddH2O, 7.5 ul 10% SDS and 10 ul tissue removal enzyme [10X Genomics, PN-300387]) on Visium CytAssist V2 slide.
28. Running Cytassist machine at 37 °C for 30 minutes.
29. Wash with 250 ul DPBS-I.
30. Add 100 ul T7 ligation mixture (50 ul 2X sticktogether buffer, 5 ul T7 DNA Ligase [NEB, M0318L], 2.5 ul RNase inhibitor and 42.5 ul ddH2O) at 25 °C 2 hr.

Reverse transcription/ Extension

1. 75 ul RT mix [10X Genomics, PN-1000189] (37.8 ul ddH_2O, 18.8 ul RT reagent, 5.2 ul TSO, 1.5 ul reducing reagent and 11.7 ul RT enzyme D) 53 °C 45 minutes.
2. Aspirate solution completely.
3. Add 150 ul 1X NEBNext polymerase mixture [NEB, M0541L] incubate 72 °C for 15 minutes.
4. Aspirate solution completely
5. Add 200 ul 50 mM Tris-HCl, 1mM EDTA, 1% SDS, and 200mM NaCl containing 0.8 ug/ml Proteinase K and incubate 58 °C for 1 hr.

6. Aspirate solution and wash with 150 ul DPBS.
7. Aspirate solution and wash with 150 ul EB buffer.
8. Aspirate solution and add 35 ul 0.08N NaOH and incubate for 10 minutes.
9. Add 5 ul 1M Tris-HCl to tubes and transfer 35 ul samples from wells to tubes.

Second strand synthesis

1. Add 75 ul second stranded synthesis solution (1.5 ul second stranded enzyme, 69.5 ul second stranded reagent, 4 ul second stranded primer) and incubate 65 °C for 15 minutes.
2. Aspirate solution and wash with 150 ul EB buffer.
3. Aspirate solution and add 35 ul 0.08N NaOH and incubate for 10 minutes.
4. Add 5 ul 1M Tris-HCl to tubes and transfer 35 ul samples from wells to tubes.

Primary ATAC-seq amplification:

PCR Amplification

5 ul	25 uM Truseq Read 1
5 ul	25 uM Nextera Read 2
50 ul	2x NEBNext Master Mix
40 ul	Sample

qPCR Amplification

3.25 ul	Sterile water
0.5 ul	25 uM Truseq Read 1
0.5 ul	25 uM Nextera Read 2
0.75 ul	20x EvaGreen
5 ul	2x NEBNext Master Mix
5 ul	Pre-Amplified Sample

Cycling Conditions

98 °C 30 sec
Then 30 cycles of:
98 °C 10 sec 63 °C 30 sec 72 °C 1 min
Hold at 10 °C

Cycling Conditions

72 °C 5 min
98 °C 30 sec
Then cycles depending on qPCR: 98 °C 10 sec 63 °C 30 sec 72 °C 1 min
Hold at 4 °C

ATAC-seq cleanup:

1. Vortex to resuspend SPRIselect reagent. Add 0.9X SPRIselect reagent to each sample.
2. Incubate 5 min at room temperature and place on the magnet stand for 5 minutes.
3. Remove supernatant and add 150µl 80% ethanol to the pellet. Wait for 30 seconds and remove the ethanol. Reap this step for a total of 2 washes.
4. Remove from the magnet. Add 21 µl Buffer EB to each sample and incubate 5 min at room temperature.
5. Place on the magnet stand for 5 minutes. Transfer 20 µl sample to a new tube strip.

Secondary ATAC-seq amplification:

PCR Amplification

5 ul	25 uM SP-Ad1 primer
5 ul	25 uM SP_ATAC Ad2
30 ul	2x NEBNext Master Mix
20 ul	Pre-amplified Sample

qPCR Amplification

2.55 ul	Sterile water
0.85 ul	25 uM Truseq Read 1
0.85 ul	25 uM Nextera Read 2
0.75 ul	20x EvaGreen
5 ul	2x NEBNext Master Mix
5 ul	Pre-Amplified Sample

Cycling Conditions

98 °C 30 sec
Then 30 cycles of:
98 °C 10 sec 63 °C 30 sec 72 °C 1 min
Hold at 10 °C

SPRIselect beads cleanup and gel extraction:

1. Vortex to resuspend SPRIselect reagent. Add 0.8X SPRIselect reagent to each sample.
2. Incubate 5 min at room temperature and place on the magnet stand for 5 minutes.
3. Remove supernatant and add 150 µl 80% ethanol to the pellet. Wait for 30 seconds and remove the ethanol. Reap this step for a total of 2 washes.
4. Remove from the magnet. Add 21 µl Buffer EB to each sample and incubate 5 min at room temperature.

5. Place on the magnet stand for 5 minutes. Transfer 20 µl sample to e-gel extraction.

RNA-seq libraries preparation:

1. Follow 10X genomics Visium Spatial Gene Expression manual. ** Change annealing temperature from 54 °C to 63 °C for Step 5.5 Sample Index PCR.
